# Digital SARS-CoV-2 Detection Among Hospital Employees: Participatory Surveillance Study

**DOI:** 10.2196/33576

**Published:** 2021-11-22

**Authors:** Onicio Leal-Neto, Thomas Egger, Matthias Schlegel, Domenica Flury, Johannes Sumer, Werner Albrich, Baharak Babouee Flury, Stefan Kuster, Pietro Vernazza, Christian Kahlert, Philipp Kohler

**Affiliations:** 1 Department of Economics University of Zurich Zurich Switzerland; 2 Clinic for Infectious Diseases and Hospital Epidemiology Cantonal Hospital St. Gallen St Gallen Switzerland; 3 Medical Research Center Cantonal Hospital St. Gallen St Gallen Switzerland; 4 Federal Office of Public Health Bern Switzerland; 5 Department of Infectious Diseases and Hospital Epidemiology Children’s Hospital of Eastern Switzerland St Gallen Switzerland

**Keywords:** digital epidemiology, SARS-CoV-2, COVID-19, health care workers

## Abstract

**Background:**

The implementation of novel techniques as a complement to traditional disease surveillance systems represents an additional opportunity for rapid analysis.

**Objective:**

The objective of this work is to describe a web-based participatory surveillance strategy among health care workers (HCWs) in two Swiss hospitals during the first wave of COVID-19.

**Methods:**

A prospective cohort of HCWs was recruited in March 2020 at the Cantonal Hospital of St. Gallen and the Eastern Switzerland Children’s Hospital. For data analysis, we used a combination of the following techniques: locally estimated scatterplot smoothing (LOESS) regression, Spearman correlation, anomaly detection, and random forest.

**Results:**

From March 23 to August 23, 2020, a total of 127,684 SMS text messages were sent, generating 90,414 valid reports among 1004 participants, achieving a weekly average of 4.5 (SD 1.9) reports per user. The symptom showing the strongest correlation with a positive polymerase chain reaction test result was loss of taste. Symptoms like red eyes or a runny nose were negatively associated with a positive test. The area under the receiver operating characteristic curve showed favorable performance of the classification tree, with an accuracy of 88% for the training data and 89% for the test data. Nevertheless, while the prediction matrix showed good specificity (80.0%), sensitivity was low (10.6%).

**Conclusions:**

Loss of taste was the symptom that was most aligned with COVID-19 activity at the population level. At the individual level—using machine learning–based random forest classification—reporting loss of taste and limb/muscle pain as well as the absence of runny nose and red eyes were the best predictors of COVID-19.

## Introduction

The COVID-19 pandemic is one of the greatest health challenges that societies around the globe have ever experienced. A range of instruments and ways to measure factors related to COVID-19 and the pandemic have been described [1-7]. COVID-19 presents a challenge for public health in general, while health care workers (HCWs) are at particular risk of acquiring COVID-19 [8]. Several studies using online forms have found they can be useful for tracking disease activity in different locations, including workplaces [9,10]. However, these technological platforms require timely, persistent, and ongoing engagement to generate valid and representative surveillance data [1]. In the context of collaboration and the collection of collective health information, digital epidemiology and participatory surveillance techniques have been demonstrated to be tools with great potential for helping to detect health threats [11-16]. Many strategies that involve daily reporting of symptoms through the voluntary participation of individuals have reported successful results [17,18]. Participatory surveillance by patients has been shown to have a complementary role in detecting syndromic clusters for several epidemiological challenges, such as COVID-19, seasonal influenza, or high-risk mass gatherings [17-22]. The implementation of novel techniques represents an additional opportunity for the rapid analysis of big data based on machine learning, thereby acting as a complement to traditional disease surveillance systems.

The objective of this work is to describe a web-based participatory surveillance strategy among HCWs in two Swiss hospitals during the first wave of the COVID-19 pandemic.

## Methods

### Study Design

A prospective cohort of HCWs was recruited in March 2020 at the Cantonal Hospital of St. Gallen and the Eastern Switzerland Children’s Hospital, Switzerland. Individuals aged 16 years and older were eligible. HCWs were enrolled in the study after accepting the electronic informed consent form. The anonymization of participants was carried out by using a management ID system with three levels; we anonymized the participants (user ID), surveys (survey ID), and their samples (order ID). No compensation was provided and participation was voluntary. A copy of the informed consent with all details about privacy and confidentiality is provided in Multimedia Appendix 1. The study was approved by the local ethics committee (Ethikkommission Ostschweiz; #2020-00502). All participants received a link via email to fill in a baseline questionnaire collecting data on pre-existing conditions at the start of the study. To improve the data quality and reduce reporting bias, mobile number validation was required; participants could only move forward if they input a token sent to their mobile phone. After completing the baseline form, participants became eligible to receive the daily SMS text message with an individualized link redirecting them to a secure web platform where they could fill in their symptom diary. To encourage participant engagement through the entire period, an SMS text message reminder was sent to those that did not fill in the symptom diary the day before. In the symptom diary, participants were asked about the type and severity of COVID-19 symptoms according to Table 1. Those that met SARS-CoV-2 testing criteria (ie, fever/feverishness, cough, shortness of breath, sore throat, or anosmia/ageusia) according to the Swiss Federal Office of Public Health (FOPH) were asked to schedule an appointment for a nasopharyngeal swab [23].

For validation purposes, the positivity rate of the online survey was compared to the positivity rate of HCWs undergoing SARS-CoV-2 polymerase chain reaction (PCR) testing at the study institutions (independent of study participation). We tested both isolated symptoms and various combinations, including the FOPH testing criteria.

**Table 1:** List of symptoms and consequences.

Survey question topic	Type
Sore throat	Symptom
Cough	Symptom
Shortness of breath	Symptom
Runny nose	Symptom
Headache	Symptom
Diarrhea	Symptom
Anorexia/nausea	Symptom
Fever	Symptom
Chills	Symptom
Limb/muscle pain	Symptom
Loss of taste	Symptom
Itchy red eyes	Symptom
Feeling weak	Symptom
Fever-related muscle pain	Symptom
Took medicines	Consequence
Sought health care	Consequence
Missed work	Consequence
Hospitalized	Consequence

### Data Analysis

For the analysis of time trends of symptoms, we used a locally weighted running line smoother (locally estimated scatterplot smoothing [LOESS]) [24], which is a nonparametric smoother with Gaussian noise added in the sine wave. This algorithm estimates the latent function in a pointwise fashion. This method is a supervised machine learning approach and was carried out to generate a moving average for scatterplot smoothing among the data points. Its function can be expressed as the following:

ω (χ)=(1–|d|^3^)^3^

where *d* is the distance of the data point from the point on the fitter curve, scaled to lie in the range from 0-1. We then used a moving average with 7 days as the window size, aligned on the right.

The Spearman rank correlation coefficient was used to verify the statistical dependence between symptoms and test positivity, using a monotonic function described by the following formula [25]:




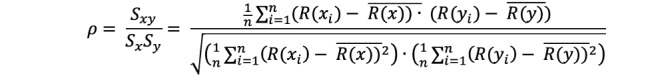




It is critical to identify significant temporal deviations throughout the period including the impact of seasonality in such high frequency data inputs. Therefore, we applied the Seasonal-Hybrid Extreme Studentized Deviate (S-H-ESD) algorithm [26], which uses a modified Seasonal-Trend decomposition procedure based on LOESS [27]. This technique allows for the identification of change points over time, recognizing when the signal frequency (FOPH classification) was positive (increasing) or negative (decreasing). The missing value was handled by spline interpolation, the maximal anomaly ratio was 0.1, and a piecewise median time window of 2 weeks was chosen.

Finally, to classify participants according to the probability of having symptoms compatible with COVID-19, we used the random forest algorithm. This is an ensemble learning method based on decision trees, which increases the accuracy of classification for both training and test data [28]. Specifically, this algorithm is a predictor consisting of an assembly of randomized base regression trees {*r_n_*(x,Θ*_m_*,*D_n_*),*m* ≥ 1}, where Θ_1_,Θ_2_,... are independent and identically distributed (IID) outputs of a randomizing variable Θ. These random trees are pooled to form the following aggregated regression estimate [29]:




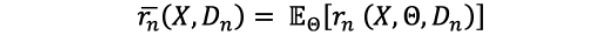




where 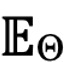 denotes expectation with respect to the random parameter, conditionally on *X* and the data set *D_n_*.

To explain how the random forest technique was used in this study, a summary of its parameters along with a prediction matrix for the model was generated. In addition, a receiver operating characteristic (ROC) curve was created to evaluate the binary classification of the model. We split the data, using 70% of entries for model training and 30% of entries for the test set. To determine which variables were more or less important for predicting the outcome, we used a boxplot chart.

Algorithms and techniques were programmed and deployed in R language, using the Exploratory [30] framework. The data collection system was developed using JotForm [31] as well as a proprietary solution and was hosted at Amazon Web Services, using EC2 and S3 instances. The SMS text messaging system used Twilio’s [32] application programming interface to send out the messages.

## Results

**Figure 1:**
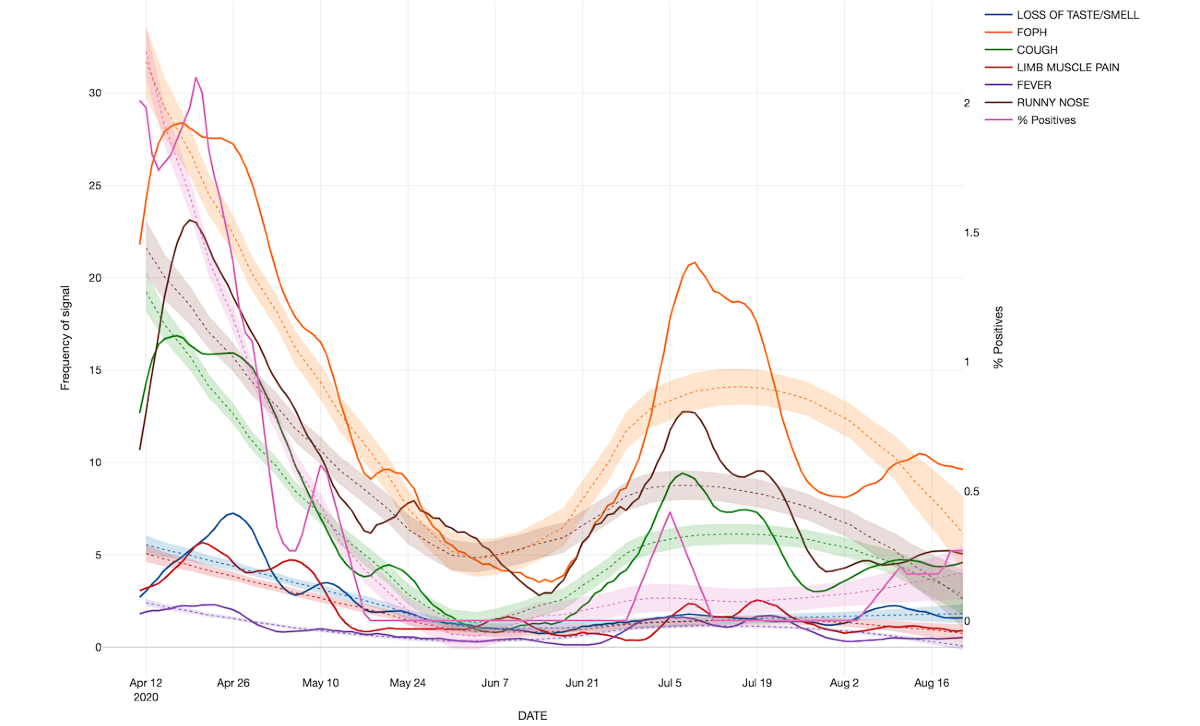
Temporal distribution and LOESS regression of symptoms related to acute respiratory infection in health care workers at two hospitals in Switzerland. FOPH: cases documented by the Federal Office of Public Health; LOESS: locally estimated scatterplot smoothing.

From March 23, 2020, to August 23, 2020, a total of 127,684 SMS text messages were sent, generating 90,414 valid reports among 1004 participants, achieving a weekly average of 4.5 (SD 1.9) reports per user. Female gender (n=755, 75.2%) was more prevalent than male (n=249, 24.8%) among participants, reflecting the general HCW population in these hospitals. The median age was 39 years, with a mean of 40.2 (SD 11.3) years. Figure 1 shows the temporal distribution of symptoms of respiratory infection over the study period, using LOESS regression. In total, 1.49% (n=15) of participants reported a positive PCR result during the study period. The first peak of the bimodal curves clearly parallels the reference curve of individuals in the hospital who tested positive, representing the first COVID-19 wave in the region. The second peak appears between July 2020 and August 2020, with a much lower signal in the reference curve of individuals who tested positive.

**Figure 2:**
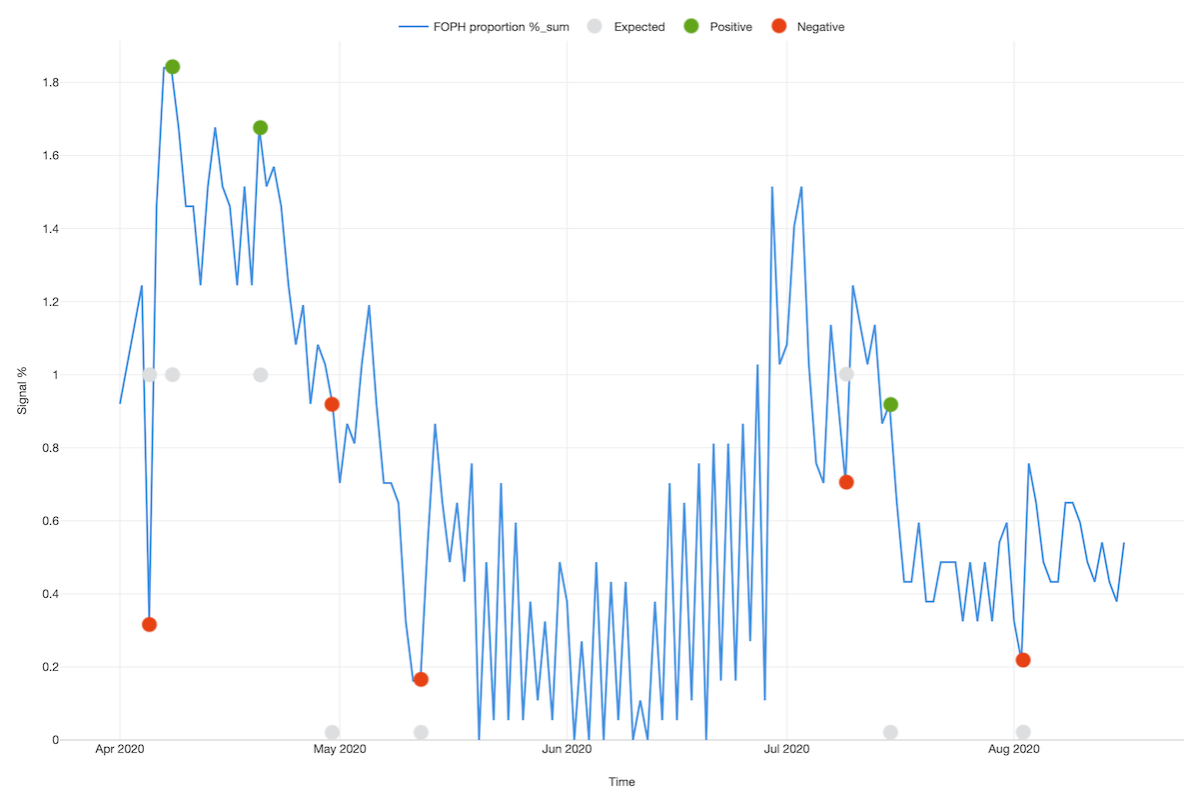
Temporal distribution of the FOPH proportion of positives, indicating which types of anomalies occurred in health care workers in two hospitals in Switzerland. FOPH: Federal Office of Public Health.

Regarding anomaly detection over time, Figure 2 shows whether a signal of symptoms was expected (based in the past trends) or if it represented a positive or negative anomaly, meaning a significant increase or decrease in the frequency of recorded symptoms. Table 2 indicates the change points that were statistically significant, including the difference observed when compared with the expected amount. The positive anomalies happened in three different periods; two of them occurred during the highest activity of the first wave and the third occurred between July and August, representing a possible second wave. However, as mentioned above, no second (or third) wave was seen in the reference curve.

**Table 2:** Significant (*P*<.05) timepoints for anomaly detection in health care workers, Switzerland.

Date	Federal Office of Public Health proportion of positives	Expected	Difference from expected	Anomaly type
05/04/2020	.324675325	1	–.675324675	Negative
08/04/2020	1.83982684	1	.83982684	Positive
20/04/2020	1.677489177	1	.677489177	Positive
30/04/2020	.91991342	0	.91991342	Negative
12/05/2020	.162337662	0	.162337662	Negative
09/07/2020	.703463203	1	–.296536797	Negative
15/07/2020	.91991342	0	.91991342	Positive
02/08/2020	.216450216	0	.216450216	Negative

**Figure 3:**
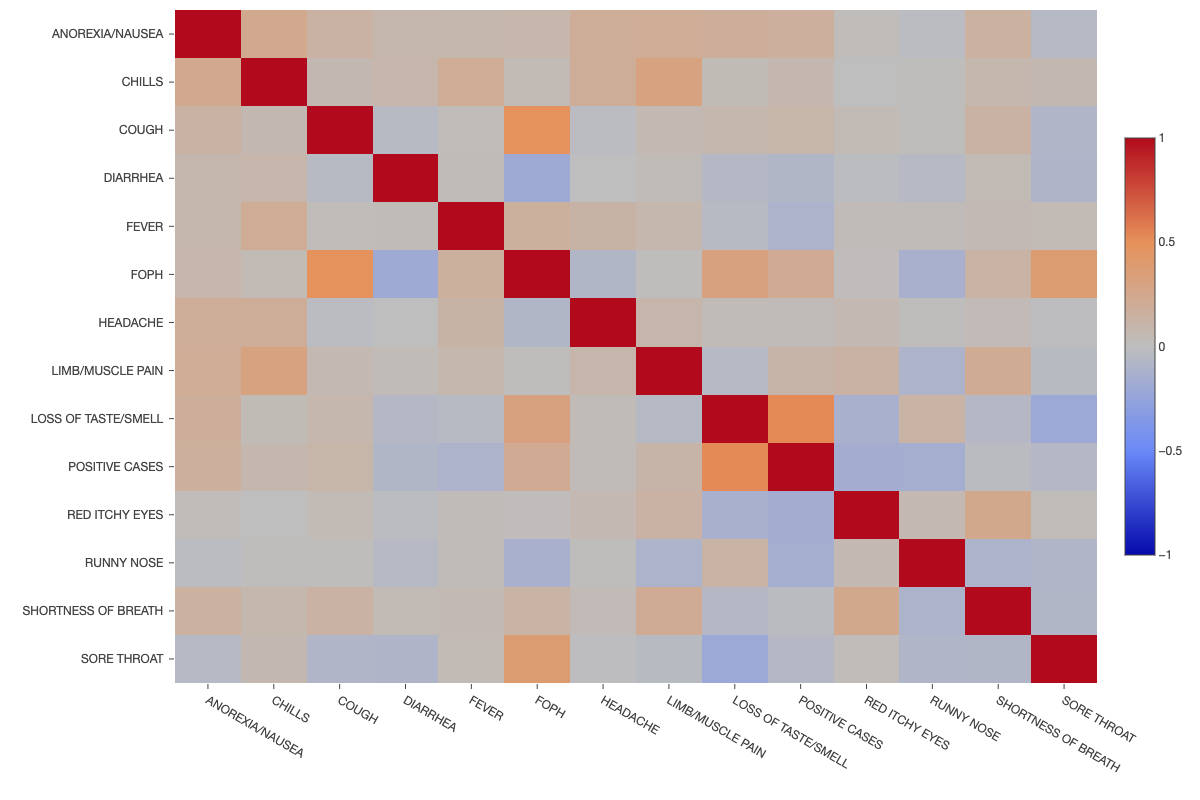
Correlation matrix using the Spearman method for symptoms and positive results in health care workers in two hospitals in Switzerland during the study period. FOPH: Federal Office of Public Health.

**Figure 4:**
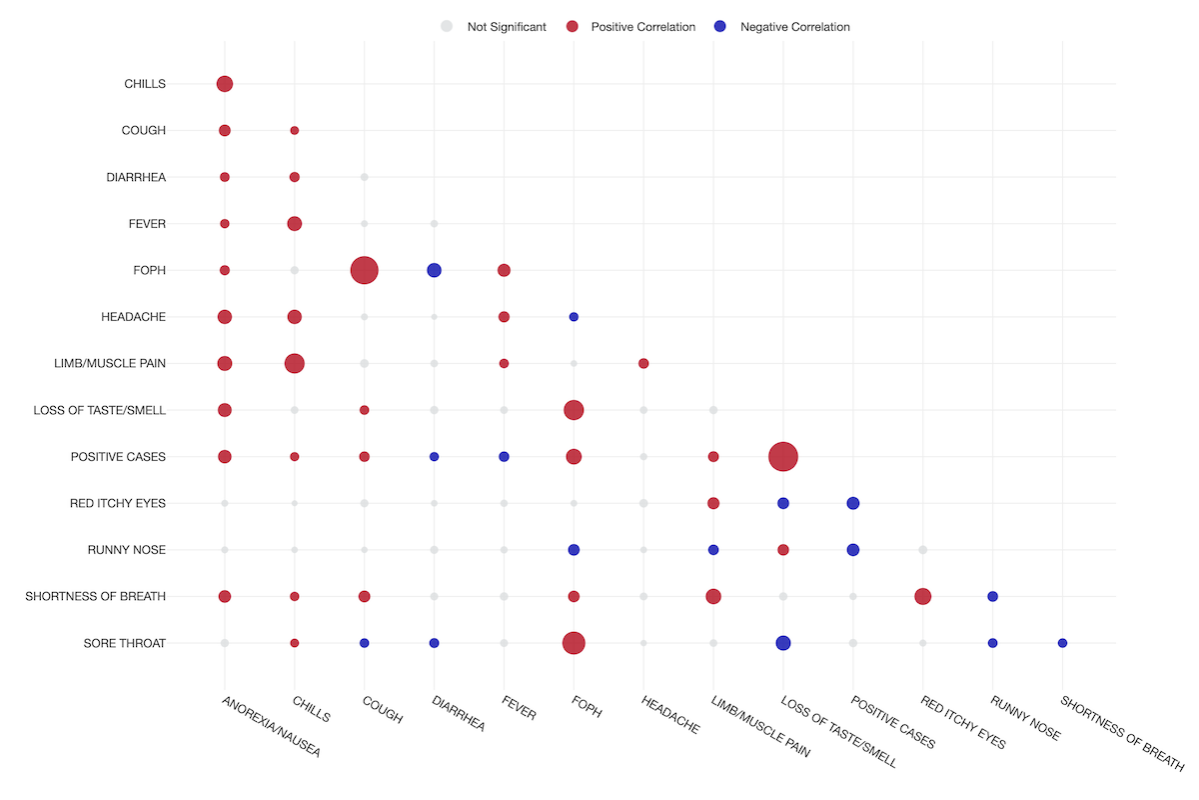
Significance matrix showcasing the positive and negative correlations between variables in health care workers in two hospitals in Switzerland during the study period. A larger dot represents a higher correlation.

A correlation matrix between symptoms and a positive PCR test result for SARS-CoV-2 is shown in Figure 3, while in Figure 4, the significance matrix shows the positive and negative correlations, as well as the nonsignificant ones. The symptom with the strongest correlation with a positive PCR result was loss of taste. Conversely, symptoms such as red eyes or runny nose were negatively associated with a positive test (Table 3).

**Table 3:** Correlation between symptoms and positive cases in health care workers in Switzerland for the period of the study.

Symptoms	Correlation	Pairs	*P* value
Loss of taste	0.5274	Positive	<.001
Federal Office of Public Health definition	0.2189	Positive	<.001
Anorexia/nausea	0.1698	Positive	<.001
Limb/muscle pain	0.1103	Positive	<.001
Cough	0.1032	Positive	<.001
Chills	0.0731	Positive	.002
Headache	0.0279	Positive	.37
Red itchy eyes	–0.1560	Negative	.01
Runny nose	–0.1508	Negative	.001
Fever	–0.1025	Negative	.10
Diarrhea	–0.0770	Negative	.001

**Figure 5:**
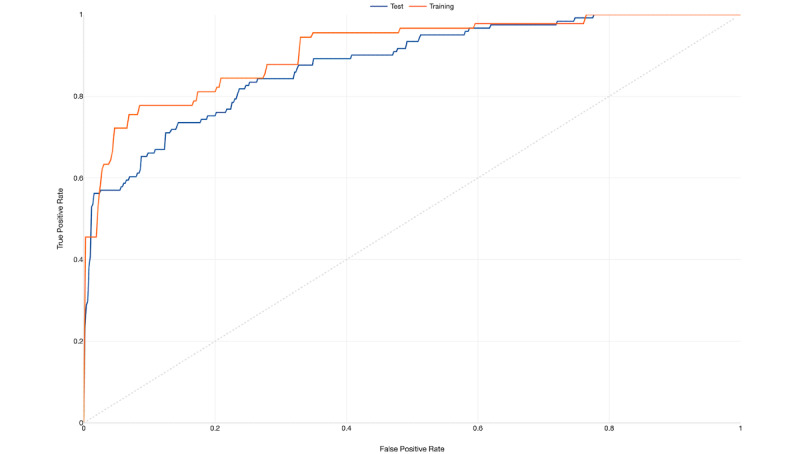
Receiver operating characteristic curve for the random forest model.

**Figure 6:**
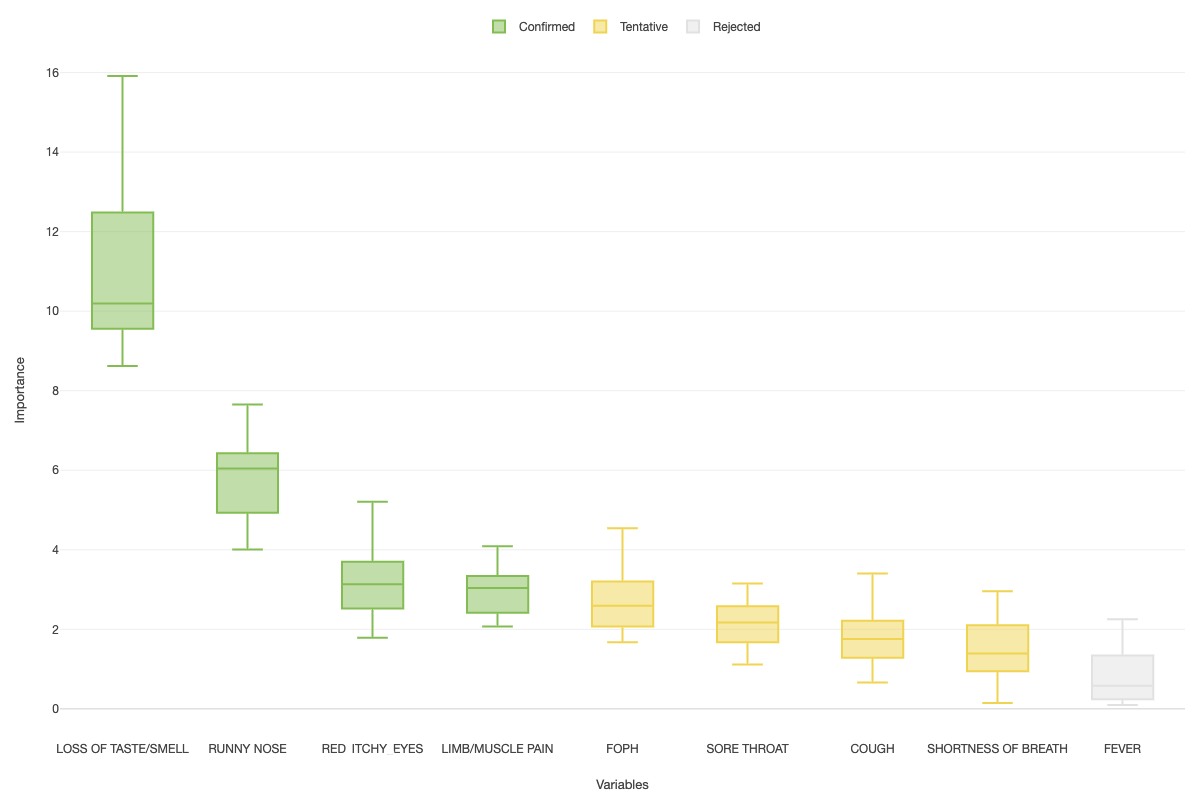
Boxplot of the importance of symptoms and their capacity to predict the expected outcome based on the random forest algorithm (*P*<.05). Loss of taste, limb/muscle pain, FOPH (Federal Office of Public Health), sore throat, cough, and shortness of breath were positively associated with the outcome. Runny nose and red itchy eyes were negatively associated with the outcome. Fever was neither positively nor negatively associated with the outcome.

Finally, Table 4 shows the summary results from a random forest algorithm that was used to classify participants into SARS-CoV-2 positive and negative cases based on their indicated symptoms. The area under the ROC curve shows reasonable performance of the classification tree, with an accuracy of 88% for the training data and 89% for the test data (Figure 5). Nevertheless, while the prediction matrix showed good specificity (80.0%), sensitivity was low (10.6%; Table 5). Figure 6 shows the importance of symptoms and their capacity to predict the expected outcome based on the random forest algorithm, considering a *P* value of <.05. Loss of taste and limb/muscle pain were the most important variables for prediction of a positive result, while runny nose and red eyes were negatively correlated with the same outcome. Fever was a very weak predictor of a positive result.

**Table 4:** Summary of the parameters of the random forest model.

Data set	Area under the curve	*F*_1_ score	Accuracy rate	Misclassification rate	Precision	Recall
Training	.90375	.68027	.8839	.11604	.87719	.5555
Test	.87576	.66331	.89438	.10561	.91304	.5206

**Table 5:** Prediction matrix for the random forest model.

Data set and type (actual)		Data type (predicted)			
		TRUE, %		FALSE, %	
**Test**					
	TRUE		10.4		9.57
	FALSE		.99		79.04
**Training**					
	TRUE		12.35		9.88
	FALSE		1.73		76.05

## Discussion

This study demonstrates the use of digital surveillance to monitor COVID-19 activity among HCWs. Loss of taste was the symptom that was most aligned with COVID-19 activity at the population level. At the individual level, using machine learning–based random forest classification, reporting loss of taste and limb/muscle pain as well as absence of runny nose and red eyes were the best predictors of COVID-19. The main strengths of the study are its high response rate and the comparison to a reference curve, which was based on documented PCR results in the same population.

Syndromic surveillance through participatory surveillance has been shown to be a feasible strategy to monitor COVID-19 activity [33], and is considered an important measure to inform the public health response to this pandemic [34]. Considering that engagement is a key element of a successful platform, our study—with an average response of 4.5 answers per week—has an excellent basis to produce valid and representative results. This high rate of engagement and participation is extraordinary when compared to other platforms [13,17,18,33], especially over a period of 5 months [35]. The easy-to-use survey, the defined population of HCWs from two different hospitals, and the regular interaction with study participants are potential reasons for this high response rate. It remains to be seen if these engagement indexes can be maintained when the study is scaled up to larger communities.

The temporal distribution of symptoms followed the trends represented in the first wave of COVID-19 in Switzerland [36,37]. However, the signals detected in July were not due to COVID-19, as shown by the reference curve. Interestingly, several HCWs tested positive for rhinovirus during this time period, suggesting that this was the reason for this wave. Of note, loss of taste, the most specific symptom of COVID-19, did not increase during this second wave.

Several other studies have shown that loss of taste is a good proxy for COVID-19 [38-41]. Although the specificity of this symptom is excellent, only about 20% of patients report loss of taste [42]. We conclude that the detection of loss of taste is very helpful to interpret findings at the population level, but less so at the individual patient level because of its low prevalence. The second most important positively associated symptom in our analysis was limb/muscle pain, which has also been noted by others [43]. Remarkably, runny nose and red eyes were very important negative predictors of COVID-19; this finding is particularly useful for when surveillance is performed during allergy season. However, both the sensitivity and specificity of a symptom depend on the background activity of other infections and allergies and might therefore be subject to change. The validity of a symptom may also change due to genetic adaptations in the dominant SARS-CoV-2 strain. During the study period, none of the new variants of SARS-CoV-2 (eg, B.1.1.7/Alpha) were circulating in Switzerland. Therefore, the symptoms described here cannot necessarily be extrapolated to a different circulating SARS-CoV-2 variant. However, syndromic surveillance through participatory surveillance may allow for the detection or validation of a different clinical presentation emerging from a new circulating strain. Indeed, a recent study describes small differences in COVID-19 symptoms in the general population in the United Kingdom depending on the variant [44].

Our study has a number of limitations. First, it was performed outside influenza season. Because influenza more often presents with constitutional symptoms than other respiratory viruses, distinguishing influenza from COVID-19 by analysis of symptoms is difficult. Second, we relied on participants self-reporting their symptoms, a method that is prone to bias. Third, generalizability of our data is limited because only one-fifth of the HCWs from our hospitals participated in the study; in addition, the spatial component could not be explored due to these same reasons. At the same time, this would be a very important parameter for evaluating whether SARS-CoV-2 is being regionally distributed, which would be useful to form a complete picture for disease surveillance purposes. The application of classification techniques based on machine learning, such as random forest classification, has its own limitations, as a large number of trees can make the algorithm too slow and ineffective for real-time predictions. In general, these algorithms are fast to train, but quite slow to create predictions once they are trained. A more accurate prediction requires more trees, which results in a slower model.

Nevertheless, we deem the presented surveillance tool highly useful in monitoring and predicting COVID-19 activity among our HCWs. Currently, we have expanded our HCW cohort to include over 5000 participants from over 20 institutions [45]. The analysis of data from different institutions will allow us to detect the clustering of cases in certain institutions, which might trigger targeted intervention measures in affected health care institutions. Additionally, these data allow for the detection of symptomatic HCWs who were either not tested or had a false-negative PCR result, and also for the discrimination of symptoms caused by SARS-CoV-2 from symptoms caused by other viruses, such as influenza. Further questions, which we aim to answer with the surveillance data generated in this larger cohort, include how long HCWs with documented SARS-CoV-2 infection (or vaccination) are protected against reinfection or how the emergence of viral variants might change the symptomatology of COVID-19.

## Appendix

Multimedia Appendix 1 Informed consent (in German).

## Abbreviations

FOPH: Federal Office of Public Health
HCW: health care worker
LOESS: locally estimated scatterplot smoothing
PCR: polymerase chain reaction
ROC: receiver operating characteristic
S-H-ESD: Seasonal-Hybrid Extreme Studentized Deviate
