# Supplementary material for: Digital SARS-CoV-2 Detection Among Hospital Employees: Participatory Surveillance Study
Source: JMIR Public Health Surveill. 2021 Nov 22;7(11):e33576. doi: 10.2196/33576 (PMC8610449; doi:10.2196/33576)
Supplement: Multimedia Appendix 1 [file publichealth_v7i11e33576_app1.pdf]

Die Ausbreitung, die Bedeutung sowie Risikofaktoren für eine Coronavirus-Infektion (COVID-19) in einer prospektiven Kohorte von Spitalmitarbeitern

(Coronavirus bei Spitalmitarbeitern)

Kantonsspital St. Gallen

Sehr geehrte Dame, sehr geehrter Herr

Wer wir sind:
Wir sind das Team der Klinik Infektiologie / Spitalhygiene am Kantonsspital St.Gallen. Zur Zeit sind wir unter dem Eindruck der sich ausbreitenden Coronavirus-Pandemie und möchten Sie mit diesem Projekt anfragen uns zu helfen, die Verbreitung dieser neuen Infektionskrankheit besser zu verstehen.

Weshalb wir Sie anfragen:
Wir möchten Sie als Mitarbeiter des KSSG und des OKS einladen, die medizinische Forschung zu unterstützen. Die zentrale Idee hinter dieser Studie gründet auf der Tatsache, dass niemand weiss, wie viele Personen in einer betroffenen Bevölkerung von einer Coronavirus-Infektion betroffen sind, ohne dass es je zu einer Infektion mit Beschwerden kommt, sogenannte asymptomatische Serokonversion. Da die Ausbreitung des Coronavirus bei uns in der Schweiz erst gerade beginnt, sind wir durch den raschen Start der Studie in der Lage, einige Fragen über die anfängliche Verbreitung der Infektion zu klären.

Um den genauen Zeitpunkt einer Infektion ohne Beschwerden zu klären, brauchen wir regelmässige Blutproben, wobei es sich nur um wenige Blutstropfen pro Zeitpunkt handelt. Dies ist der Grund, weshalb wir uns mit diesem Projekt an Beschäftigte im Gesundheitswesen richten. Einerseits wissen wir, dass auch Sie die Bedeutung der hier untersuchten Frage gut kennen. Fast wichtiger noch: Sie sind in der Lage, sich selbst eine kapillare Blutentnahme zu entnehmen oder haben engen Kontakt zu KollegInnen, die dies bei Ihnen durchführen können. Zusätzlich werden wir während der Studie Nasenabstriche durchführen.

Wer darf mitmachen?
Alle Mitarbeiter im KSSG sowie im OKS, welche mindestens 16 Jahre alt sind dürfen bei dieser Studie mitmachen. Patientenkontakt ist nicht notwendig für eine Teilnahme.

Ablauf und Inhalt Forschungsprojekt

Studieneinschluss und Blutentnahme
Jede Teilnehmerin/jeder Teilnehmer macht für den Studieneinschluss einen Termin im infektiologischen Ambulatorium ab für die Einwilligung sowie die Abgabe der Studienmaterialien. **Ausserdem machen wir bei Ihnen bei dieser Gelegenheit eine venöse Blutentnahme.** Der Grund hierfür ist, dass wir nebst der zentralen Frage der stillen Serokonversion auch weiteren Fragen nachgehen. Insbesondere interessiert uns, was für Risikofaktoren (klinische, epidemiologische, hygienische) mit einer Erkrankung assoziiert sind. Weiter wissen wir von anderen Viren, dass viele Menschen gar nie infiziert werden, weil sie eine genetisch vermittelte Resistenz gegen das Virus haben. Es ist durchaus möglich, dass eine solche genetische Resistenz auch für das COVID-19 vorhanden ist und mögliche Kandidaten eines solchen genetischen Schutzmechanismus in den nächsten Monaten identifiziert werden. Wenn möglich möchten wir auch eine solche genetische Resistenz gegenüber COVID-19 untersuchen. Weiter werden wir auch andere Faktoren, die mit einem erhöhten Risiko für COVID-19 assoziiert sein können, untersuchen, wie zum Beispiel erhöhter Nachweis von gewissen Eiweissen im Blut (z.B. Furin).

2-wöchentlicher Fingerpieks
Im Zentrum der Studie steht die Frage der COVID-19 Serokonversion, das heisst die Frage, wie häufig Mitarbeiter im Gesundheitswesen zu Beginn der Ausbreitung der COVID-19 Pandemie eine positive Antikörperantwort entwickeln und wie oft diese «Serokonversion» ohne Krankheitszeichen abläuft. **Wir werden Sie daher bitten, einmal alle 2 Wochen einige Tropfen Blut auf einem Filterpapierträger zu asservieren und uns dieses zukommen zu lassen**. Dies kann auch eine Kollegin/ein Kollege bei Ihnen durchführen. Für die korrekte Entnahme lesen Sie bitte das entsprechende Merkblatt und schauen Sie sich das Video auf infekt.ch/studie an.
Die Filterpapierproben aus dem Kapillarblut werden in einer Aluminiumfolie verschweisst und die Proben mit einem eindeutigen Barcode, den Sie von uns erhalten, bezeichnet. Die vorbeschrifteten Proben senden Sie per interner Post oder Rohrpost an die Studienkoordination. Bis zur Durchführung der geplanten Analyse werden die Filterproben dann tiefgefroren gelagert. Die Studie ist so geplant, dass im Rahmen der prospektiven Erfassung laufend Blutproben gesammelt werden und die Analyse dieser Proben dann zu einem späteren Zeitpunkt erfolgt. Dies erlaubt uns durch einen vertretbaren finanziellen Aufwand heute schon Proben zu sammeln, bevor die vollständige Finanzierung der in Zukunft geplanten Labortests gesichert ist, was für den geforderten raschen Beginn der Studie zwingend ist.
Wir möchten uns vorbehalten, Sie am Ende der Studie für eine zusätzliche venöse Blutprobe aufzubieten, sollten die serologischen Tests ab Filterpapier qualitativ ungenügend sein.

Nasenabstrich bei Symptomen
Wir möchten auch versuchen, eine Infektion mit einem direkten Virusnachweis nachzuweisen. Daher werden wir Sie bitten, sollten Sie Symptome einer grippalen Erkrankung aufweisen, sich beim Personalärztlichen Dienst (PAD) zu melden, damit ein Nasenabstrich gemacht werden kann, welcher auf SARS-CoV-2 (RNA-Nachweis mittels PCR) untersucht wird. Je nach Symptomen, welche Sie im täglichen SMS angeben (siehe unten), werden wir Sie direkt auffordern, beim PAD einen Termin für diesen Abstrich zu vereinbaren (Telefon 071 494 12 03). Dieses Resultat erfahren Sie direkt nach Testung.

Einmaliger Nasenabstrich während Peak der Epidemie
Zum Zeitpunkt des Maximums der Epidemie (geschätzt April 2020) werden wir während einer ganzen Woche bei möglichst vielen Studienteilnehmern einen Nasenabstrich bei uns im Ambulatorium durchführen, unabhängig von Ihren Symptomen. Sie werden diesbezüglich ein Aufgebot per Email UND SMS erhalten. Je nach Finanzierung werden wir am Ende der Studie auch von diesen Abstrichen eine PCR bezüglich SARS-CoV-2 durchführen.

Online Fragebögen
Wir werden Ihnen während der Dauer der Studie regelmässig Fragebögen per Email oder SMS zukommen lassen. Als Erstes brauchen wir Angaben zu Ihrer Person, Gesundheitsdaten, Informationen zu Ihrer Arbeits- und Wohnsituation sowie zu Ihrem Hygieneverhalten inner- und ausserhalb des Spitals. **Diese Ausgangs-Daten können Sie selbständig auf einer gesicherten Online-Plattform eingeben (Aufwand einmalig ca. 15 min).** Weiter werden wir Sie zwei-wöchentlich über mögliche Expositionen (also Kontakte mit vermuteten oder bestätigten COVID-19 Fällen) sowie Ihr Hygieneverhalten fragen. **Auch dies können Sie auf der Online-Plattform ausfüllen (Aufwand alle 14 Tage ca. 10min).** Um den klinischen Verlauf einer allfälligen Erkrankung zu charakterisieren, werden wir Sie bitten, uns täglich mittels SMS mitzuteilen, ob Sie Symptome eines viralen Infektes haben oder nicht. **Im Falle von Symptomen werden Sie einen persönlichen Link auf die erwähnte Online-Plattform erhalten, wo Sie uns kurz die Details Ihrer Beschwerden**

schildern können (Aufwand jeweils ca. 5min). Zusätzlich werden Sie eventuell für einen Nasenabstrich beim Personalärztlichen Dienst eingeladen (siehe oben). Dies erlaubt uns später, nicht nur die geschilderten Symptome mit einer Serokonversion in Verbindung zu setzen. Wir können auch die Häufigkeit und Art von Symptomen im Zusammenhang mit einer COVID-19 Infektion sowie mögliche Ansteckungsquellen erfassen.

Studienbeginn und -dauer
Wir möchten mit dieser Studie rasch möglichst (Ende März 2020) beginnen, weil wir davon ausgehen, dass sehr viele Personen bereits in wenigen Wochen Antikörper gegen die Infektion mit dem Coronavirus COVID-19 aufweisen könnten. Die Dauer der Studie ist auf vier Monate limitiert. Sollten weniger Infektionen als erwartet auftreten, würden wir die Studie allenfalls um maximal drei Monate verlängern.

Screening von Haushaltkontakten
Am Ende der Studie werden wir Sie im Rahmen einer Unterstudie möglicherweise fragen, ob Ihre Haushaltskontakte bereit wären, uns ebenfalls einen Blutstropfen zur Verfügung zu stellen sowie einen kurzen Fragebogen online auszufüllen. Dies würde uns helfen abzuschätzen, wie gross der Einfluss von kranken Haushaltskontakten auf Ihr persönliches Erkrankungsrisiko ist. Ein separates Informationsschreiben mit Einwilligungsformular wird diesbezüglich noch folgen.

Ihre Rechte als Spenderin/Spender
- Sie geben nur dann Ihre Daten und Proben für die Verwendung in diesem Forschungsprojekt frei, wenn Sie es wollen.
- Sie müssen nicht begründen, wenn Sie Ihre Daten und Körpermaterialien nicht der Forschung zur Verfügung stellen möchten.
- Wenn Sie sich zur Teilnahme entscheiden, können Sie diesen Entscheid jederzeit wieder zurücknehmen. Sie müssen diesen Entscheid nicht begründen. Wenden Sie sich dazu an eine Person des Forschungsteams.
- Falls Sie sich während der Studie entschliessen Ihr Einverständnis zu widerrufen, werden alle – falls Sie dies wünschen - bis dahin gesammelten Proben entsorgt. Sind zum Zeitpunkt Ihres Widerruf bereits Proben untersucht worden, werden Ihre Daten und bestehenden Proben für das vorliegende Forschungsprojekt bei Rücktritt noch analysiert und dann anonymisiert aufbewahrt.
- Sie dürfen jederzeit alle Fragen zum Forschungsprojekt stellen, die Sie interessieren. Wenden Sie sich dazu bitte an die Person, die am Ende dieses Aufklärungsdokuments genannt ist. Sie können sich auch an jede andere Person wenden, die am Projekt mitarbeitet.

Für Sie wichtige Forschungsergebnisse
Ergebnisse aus dem Forschungsprojekt, die Sie und Ihre Gesundheit betreffen, teilen wir Ihnen gerne mit, sofern dies möglich ist. Sie können aber auch auf diese Mitteilung verzichten, wenn Sie diese Ergebnisse nicht wissen wollen. Das Wissen über Befunde aus genetischen Personendaten kann beispielsweise ein Nachteil sein, wenn Sie eine Zusatzversicherung abschliessen wollen. Bitte teilen Sie der untenstehenden Kontaktperson mit, falls Sie nicht informiert werden wollen.

Viele Ergebnisse werden keine Bedeutung für Sie als Person haben und keine Schlussfolgerung auf Ihre Gesundheit oder Ihre Behandlung zulassen. In diesen Fällen werden Sie nicht informiert.

Datenschutz
Wir behandeln alle Ihre Daten und Proben streng vertraulich. Für die Speicherung der Daten wird der Amazone Server in Frankfurt benützt. Wie bereits oben ausgeführt liegen diese in verschlüsselter Form vor. Es werden nur diejenigen Personen Zugang zu Ihren identifizierenden Angaben haben, welche diese im Rahmen des Forschungsprojekts zwingend benötigen. In der Regel ist das nur die Projektleitung. Für alle anderen Mitarbeiter werden Ihre Daten und Proben nur in verschlüsselter Form für das Forschungsprojekt zur Verfügung stehen, d.h. diese Personen wissen nicht, dass die Daten und Proben von Ihnen stammen.
Ohne Ihre Erlaubnis geben wir Ihre Daten und Proben an niemanden weiter.

Finanzierung des Forschungsprojekts
Die Startphase des Forschungsprojekt wird durch das KSSG finanziert. In dieser Finanzierung ist der Aufbau der Probensammlung (auf Filterpapier) und die Entwicklung der Online-Plattform enthalten. Die Finanzierung der Bestimmung von Antikörpern aus den tiefgefrorenen Proben, die allenfalls durchzuführende Erregeranalyse aus Nasensekret und die genetische Analyse von Resistenzfaktoren gegenüber einer COVID-Erkrankung muss vorgängig durch weitere Finanzierung gesichert werden. Wir werden im Laufe des März 2020 ein Forschungsprojekt im Rahmen einer Coronavirus-Forschungsausschreibung einreichen.

Kontaktperson
Wenn Sie weitere Fragen zu unserem Projekt haben, wenden Sie sich bitte an
E-mail: bistdudabei@kssg.ch oder

- Simone Kessler, Studienkoordination, 071 494 26 62
- PD Dr. med. Philipp Kohler, KSSG, 071 494 11 48
- Dr. med. Christian Kahlert, OKS, 078 852 02 74

Sie können sich auch an jeden anderen Mitarbeitenden des Projektes wenden.

Einwilligungserklärung zu einem Forschungsprojekt
Ich willige hiermit ein, dass meine persönlichen Daten und Daten, die ich über die studienspezifische Online-Plattform selbst eingegeben habe, sowie genetische Personendaten und Proben in verschlüsselter Form für das unten genannte Forschungsprojekt verwendet werden dürfen.

**BASEC-Nummer** (nach Einreichung):	2020-00502
**Titel des Projekts** (wissenschaftlich und in Laiensprache):	*Coronavirus bei Spitalmitarbeitern*
**verantwortliche Institution** (Projektleitung mit Adresse):	Kantonsspital St.Gallen, vertreten durch PD Dr. Philipp Kohler, Klinik Infektiologie / Spitalhygiene, KSSG, 9007 St. Gallen
**Ort der Durchführung**:	Kanton St. Gallen
**Leiter / Leiterin des Projekts am Studienort:** Name und Vorname in Druckbuchstaben	Philipp Kohler
**Teilnehmerin/Teilnehmer:** Name und Vorname in Druckbuchstaben: Geburtsdatum:	☐ weiblich ☐ männlich

Ich bestätige, dass ich
- das zu dieser Einwilligungserklärung gehörende Aufklärungsdokument erhalten habe;
- hinreichend über die Aufbewahrung und Weiterverwendung von meinen genetischen und nicht-genetischen Personendaten und Proben für das genannte Forschungsprojekt in verschlüsselter Form aufgeklärt worden bin;
- die Möglichkeit hatte, Fragen zu stellen und diese zu meiner Zufriedenheit beantwortet werden konnten;
- darüber informiert worden bin, dass meine Einwilligung freiwillig ist und mir insbesondere keine Vor- oder Nachteile entstehen, wenn ich zustimme oder ablehne;
- weiss, dass ich diese Einwilligung jederzeit widerrufen (das heisst zurückziehen) kann und dass ich keine Gründe dafür angeben muss;
- weiss, dass ich über für mich relevante Ergebnisse informiert werde. Sollte ich dies nicht wünschen, informiere ich die oben genannte Kontaktperson;
- weiss, was bei meinem Widerruf mit meinem bis dahin verwendeten Material und meinen Daten geschieht.
-

Ort, Datum — Rechtsgültige Unterschrift: Teilnehmer

Ort, Datum — Name und Vorname, rechtsgültige Unterschrift der für die Aufklärung verantwortlichen Person:
